# LOXL1 Is Regulated by Integrin α11 and Promotes Non-Small Cell Lung Cancer Tumorigenicity

**DOI:** 10.3390/cancers11050705

**Published:** 2019-05-22

**Authors:** Cédric Zeltz, Elena Pasko, Thomas R. Cox, Roya Navab, Ming-Sound Tsao

**Affiliations:** 1 Princess Margaret Cancer Center, University Health Network, Toronto, ON M5G 1L7, Canada; cedric.zeltz@uhnresearch.ca (C.Z.); elena.pasko@alum.utoronto.ca (E.P.); Roya.Navab@uhnresearch.ca (R.N.); 2 The Kinghorn Cancer Centre, Garvan Institute of Medical Research, 370 Victoria St, Darlinghurst, Sydney, NSW 2010, Australia; t.cox@garvan.org.au; 3 St Vincent’s Clinical School, UNSW Sydney, Sydney, NSW 2052, Australia; 4 Department of Laboratory Medicine and Pathobiology, University of Toronto, Toronto, ON M5S 1A8, Canada; 5 Department of Medical Biophysics, University of Toronto, Toronto, ON M5G 1L7, Canada

**Keywords:** lysyl oxidase, integrin α11, lung adenocarcinoma, matrix cross-linking, tumor progression

## Abstract

Integrin α11, a stromal collagen receptor, promotes tumor growth and metastasis of non-small cell lung cancer (NSCLC) and is associated with the regulation of collagen stiffness in the tumor stroma. We have previously reported that lysyl oxidase like-1 (LOXL1), a matrix cross-linking enzyme, is down-regulated in integrin α11-deficient mice. In the present study, we investigated the relationship between LOXL1 and integrin α11, and the role of LOXL1 in NSCLC tumorigenicity. Our results show that the expression of LOXL1 and integrin α11 was correlated in three lung adenocarcinoma patient datasets and that integrin α11 indeed regulated LOXL1 expression in stromal cells. Using cancer-associated fibroblast (CAF) with either a knockdown or overexpression of LOXL1, we demonstrated a role for LOXL1 in collagen matrix remodeling and collagen fiber alignment in vitro and in vivo in a NSCLC xenograft model. As a consequence of collagen reorganization in NSCLC tumor stroma, we showed that LOXL1 supported tumor growth and progression. Our findings demonstrate that stromal LOXL1, under regulation of integrin α11, is a determinant factor of NSCLC tumorigenesis and may be an interesting target in this disease.

## 1. Introduction

The tumor microenvironment (TME) plays an active role in non-small cell lung cancer (NSCLC) development and progression. Cancer-associated fibroblast (CAF) is a major TME cellular component, playing key roles in NSCLC tumorigenicity [1]. CAFs contribute to extracellular matrix remodeling and desmoplasia, a prognostic marker for relapse in NSCLC patients [2]. Collagen matrix remodeling and cross-linking are critical factors in tumor progression and metastasis that involve integrins, matrix-metalloproteinases (MMPs), and lysyl oxidases (LOX) [3].

The LOX family consists of five homologous members: LOX and LOX-like (LOXL) 1–4. They are secreted copper-dependent amine oxidases that catalyze covalent cross-linking of collagen and elastin fibers [4]. Lox knockout mice die perinatally due to cardiovascular dysfunction and connective tissue disorders [5,6]. Loxl1-deficient mice are viable but develop pelvic organ prolapse [7]. Loxl2 and Loxl3 knockout mice also display tissue homeostasis disorders [8,9]. The role of LOX and LOXL2 has been well characterized in human cancers [10,11,12]. LOX plays an essential role in forming a pre-metastatic niche, creating a more favorable environment for the colonization of cancer cells at distant sites [13,14]. Therapeutic approaches targeting LOXL2, by small molecule inhibitors [15] or blocking antibodies [16], have demonstrated efficacy in fibrosis and cancer models. In contrast, relatively little is known about the role of LOXL1 in cancers. Lkb1-deficient mice display enhanced metastasis and Loxl1 is among a set of genes that are up-regulated in these mice [17]. In addition, LOXL1 expression is associated with chemotherapy resistance in NSCLC and pancreatic ductal carcinoma [18,19].

We recently showed that integrin α11 promotes tumor growth and metastasis of NSCLC and these were strongly associated with the regulation of collagen stiffness in the tumor stroma [20]. Moreover, in the same study, we observed that the matrix cross-linking enzyme Loxl1 was down-regulated in the xenograft tumors of integrin α11-deficient compared to wild-type severe combined immune deficient (SCID) mice. These results provided a rationale to investigate the role of LOXL1 on lung tumor progression. Our results show that LOXL1, through regulation of integrin α11 in CAFs, mediates collagen fiber alignment in NSCLC stroma to support tumor progression.

## 2. Results

### 2.1. Integrin α11 Mediates LOXL1 Expression in Fibroblast

**Figure 1:**
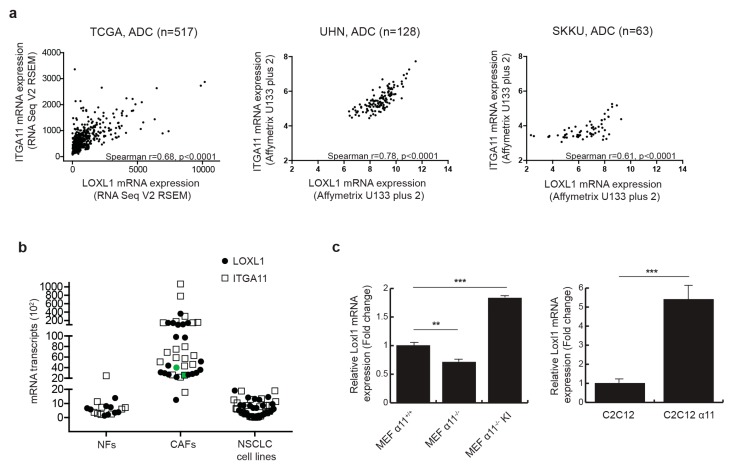
Integrin α11 regulates lysyl oxidase-like1 (LOXL1) mRNA expression. (**a**) Integrin α11 (ITGA11) and LOXL1 gene expression are strongly correlated in three independent non-small cell lung cancer (NSCLC) datasets. Spearman correlation between ITGA11 and LOXL1 gene expression was performed using RNA Seq data of The Cancer Genome Atlas (TCGA) lung adenocarcinoma dataset (*n* = 517), gene expression microarray dataset from University Health Network (UHN, *n* = 128, GSE50081) and Sungkyunkwan University (SKKU, *n* = 63, GSE8894). (**b**) ITGA11 and LOXL1 mRNA expression was analyzed using RT-qPCR and showed expression in cancer-associated fibroblasts (CAFs, *n* = 20) but not in lung normal fibroblasts (NFs, *n* = 9) or in established NSCLC cell lines (*n* = 30). CAF094 that has been used in further experiments is highlighted in green (**c**). LOXL1 expression depends on ITGA11 expression. LOXL1 expression has been studied by RT-qPCR (left panel) in wild-type mouse embryonic fibroblast (MEF) (MEF α11^+/+^), integrin α11 knockout MEF (MEF α11^−/−^), and integrin α11 knock-in MEF (MEF α11^−/−^ KI) and (right panel) in wild-type C2C12 cells and C2C12 cells expressing integrin α11 (C2C12 α11) (*n* = 3 per condition, **, *p* < 0.01; ***, *p* < 0.001; mean ± SD).

We previously reported that Loxl1 expression was down-regulated in integrin α11-deficient mouse tumor stroma [20], suggesting that integrin α11 may regulate LOXL1 expression. We first explored the clinical relevance of integrin α11 (ITGA11) and LOXL1 gene expression in NSCLC. In a lung adenocarcinoma dataset from The Cancer Genome Atlas (TCGA), we observed a strongly significant correlation between ITGA11 and LOXL1 expression (Spearman *r* = 0.68, *p*-value < 0.0001), which we further validated in two other independent patient tumor datasets (Figure 1a). In contrast, the correlation in gene expression between ITGA11 and LOXL1 in lung squamous cell carcinoma (Figure S1) and between ITGA11 and other LOX family members (Table S1) was not consistent in these three independent datasets. Integrin α11 is known to be specifically expressed in NSCLC CAFs [21]. Using RT-qPCR on a panel of nine lung normal fibroblasts (NFs), 20 human lung cancer-derived primary CAFs, and 30 established NSCLC cell lines, we confirmed that ITGA11 is expressed as a stromal gene, and further showed that LOXL1, like ITGA11, is primarily expressed in CAFs but not in tumor cells or NFs (Figure 1b). To determine the role of integrin α11 in the regulation of LOXL1 gene expression, mouse embryonic fibroblasts (MEFs) were isolated from Itga11 knockout (α11^−/−^) and wild-type mice (α11^+/+^) [22]. In the absence of integrin α11 in MEFs, there was a significant decrease (−30%, *p* < 0.01) in Loxl1 gene expression (Figure 1c). In contrast, reintroduction of ITGA11 into α11^−/−^ MEFs (MEF α11^−/−^ KI) resulted in Loxl1 overexpression (+160%, *p* < 0.001). We validated this result using the C2C12 cell line that does not express any collagen-binding integrins. The overexpression of integrin α11 in C2C12 (C2C12 α11) also led to Loxl1 overexpression (+440%, *p*-value < 0.001). These results suggest that integrin α11 regulates LOXL1 gene expression in CAFs.

### 2.2. LOXL1 Mediates Collagen Fiber Alignment in NSCLC

**Figure 2:**
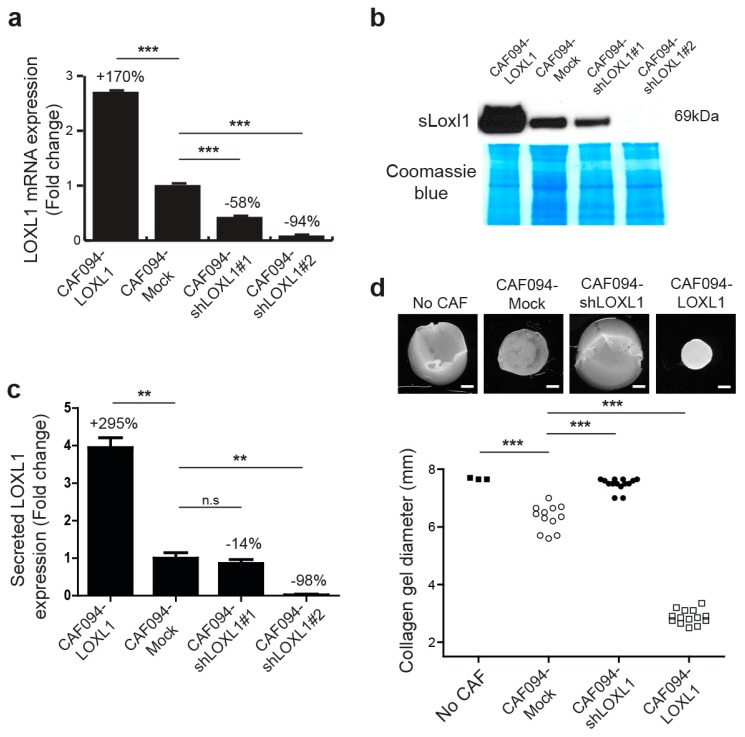
LOXL1 is required for efficient collagen matrix reorganization. CAF094^YFP-hTERT^ were infected using lentiviral strategy, either with the full length LOXL1 cDNA (LOXL1), LOXL1 short hairpin RNA constructs (shLOXL1#1 and shLOXL1#2), or with empty vector (mock). (**a**) LOXL1 gene expression was measured by RT-qPCR and the values were normalized using a housekeeping gene RPS13 (*n* = 3, ***, *p* < 0.001; mean ± SD). (**b**) The levels of LOXL1-secreted protein (sLoxl1) were analyzed by Western blot anaylsis. The full-size Western blot is displayed in Supplementary Figure S4. (**c**) The sLoxl1 band intensity was normalized to Coomassie blue staining of the developed membrane (*n* = 3, **, *p* < 0.01; n.s, not significant; mean ± SD). (**d**) Effect of LOXL1 overexpression and knock-down on collagen remodeling. CAF094^YFP-hT^ cell lines were embedded in attached collagen gel and collagen gel diameter was measured 12 days later. Acellular collagen gels were used as negative control (no CAF). Collagen gel contraction was assessed photographically. Scale bar: 1.5 mm. Statistics were performed by ANOVA followed by Bonferroni post-test (*n* = 3–14, ***, *p* < 0.001). n.s., not significant.

The prototypical LOX has been reported to be involved in matrix remodeling during breast tumorigenicity by linearizing collagen lattices [3]. We investigated whether collagen fibers were remodeled in a similar manner by LOXL1. For this purpose, we generated CAF cell lines with LOXL1 overexpression (CAF094-LOXL1) or knockdown, the latter using two short hairpin RNA (shRNA) (CAF094-shLOXL1#1 and CAF094-shLOXL1#2). Empty vector-infected cells (CAF094-Mock) were used as control. We observed a three-fold increase of *LOXL1* gene expression in CAF094-LOXL1 compared to CAF094-Mock and a decreased expression of 58% and 94% in CAF094-shLOXL1#1 and CAF094-shLOXL1#2, respectively (Figure 2a). We also confirmed the altered LOXL1 expression at the secreted protein level, except in CAF094-shLOXL1#1, in which knockdown was not significant (Figure 2b,c). Therefore, we have excluded this cell line from further experiments.

To determine whether LOXL1 affects matrix remodeling, CAFs were embedded in attached collagen gel and were cultured for 12 days (Figure 2d). In this model, the extent of gel contraction reflects the degree of matrix remodeling [23]. Knockdown of LOXL1 significantly inhibited matrix remodeling compared to CAF094-Mock (−88%, *p*-value < 0.001), displaying similar contraction as acellular collagen gels (no CAF). In contrast, LOXL1 overexpression strongly enhanced collagen matrix remodeling compared to CAF094-Mock (+284%, *p*-value < 0.001). In addition, we overexpressed LOXL1 in C2C12 cells, which lacked integrin α11, and performed collagen gel contraction assay (Figure S2). As previously published [24], C2C12-α11 cells displayed more collagen contraction compared to wild-type C2C12 cells. Interestingly, overexpression of LOXL1 in C2C12 cells rescued the reduced collagen matrix reorganization, induced by the absence of integrin α11.

**Figure 3:**
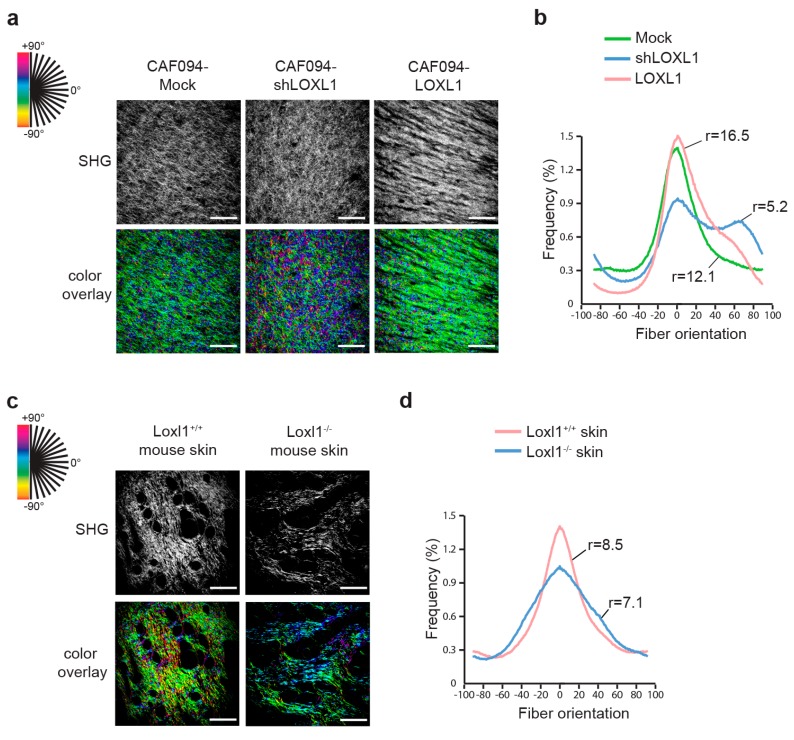
LOXL1 mediates collagen fiber alignment. Effect of LOXL1 on collagen fiber alignment has been evaluated using confocal second harmonic generation (SHG) on the CAF-populated collagen matrix (**a**,**b**) and on mouse skin dermis (**c**,**d**). SHG pictures were analyzed using OrientationJ (**a**,**c**). Each oriented structure within the image is colored according to its angle. Dominant color represents dominant alignment direction. Scale bar: 100 µm. Distribution of collagen fiber orientation within the different conditions was shown in (**b**,**d**). Peaks of distribution were aligned and the ratio between the peak and baseline of each curve was calculated to give the degree of alignment. Statistics were performed using Fisher’s test (*n* = 9 per condition, see Tables S2 and S3).

Alignment of collagen fibers in the remodeled matrix was recorded using second harmonic generation and analyzed with OrientationJ. In this model, each oriented structure within the image is colored according to its angle; a dominant color in the overlay picture thus denotes higher alignment of the collagen fibers (Figure 3a). In parallel, peaks of distribution of collagen fiber orientation were aligned and the ratio between the peak and baseline of each curve was calculated to give the degree of alignment (Figure 3b and Table S2). We showed more aligned collagen in matrices populated with CAF094-LOXL1 compared to matrices populated with CAF094-Mock (*r* = 16.5 and *r* = 12.1, respectively; *p* = 0.0013). In contrast, collagen matrices populated with CAF094-shLOXL1 displayed poor organization (*r* = 5.2; *p* < 0.0001). To confirm the role of LOXL1 in collagen fiber alignment in vivo, we collected skin tissues from Loxl1 knockout and wild-type SCID mice and we analyzed collagen organization in these tissues using the same method. Loxl1 knockout mice displayed more disorganized dermal collagen matrix compared to wild-type SCID mice (*r* = 7.1 and *r* = 8.5, respectively; *p* < 0.001; Figure 3c,d and Table S3), confirming that LOXL1 takes part in collagen organization and fiber alignment.

### 2.3. LOXL1 Induces NSCLC Cell Invasion

**Figure 4:**
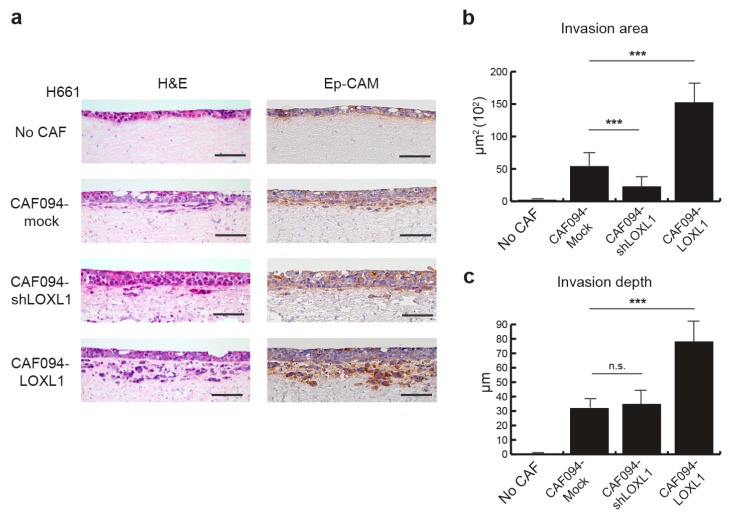
LOXL1 promotes NSCLC invasion. (**a**) Invasion of H661 lung tumor cells was studied in an organotypic model in the presence of CAFs in which LOXL1 is overexpressed (CAF094-LOXL1) or knocked down (CAF094-shLOXL1). Acellular matrix (no CAF) and matrix populated with mock-CAF (CAF094-mock) were used as control. To visualize tumor cell invasion, H661 cells were stained using an epithelial cell adhesion molecule (Ep-CAM) antibody. Scale bar: 100 µm. Quantification of H661 invasion area (**b**) and invasion depth (**c**) in the matrix was performed using imageJ (*n* = 12 per condition, ***, *p* < 0.001; n.s., not significant; mean ± SD).

Since collagen matrix remodeling and alignment have been shown to mediate metastasis [25], we investigated the role of LOXL1 in tumor cell invasion. We designed an organotypic model in which the H661 NSCLC cell line was seeded on top of a collagen–matrigel matrix populated with CAFs of varying levels of LOXL1. Acellular matrix (no CAF) was used as control. After three weeks following tumor cell attachment to the matrix, the organotypic cultures were collected and analyzed. Tumor cells were stained with an Ep-CAM antibody to visualize invasion (Figure 4a). We did not observe any invading tumor cells in the absence of CAFs, indicating that CAFs are required for tumor cell invasion. Overexpression of LOXL1 in this model (CAF094-LOXL1) significantly enhanced the number and depth of tumor cell invasion compared to the invasion in CAF094-Mock-populated matrices (*p* < 0.001; Figure 4b,c). Furthermore, LOXL1 knockdown decreased tumor cell invasion (*p* < 0.001), confirming the role of LOXL1 in NSCLC cell invasion.

### 2.4. LOXL1 Promotes In Vivo Tumor Growth

**Figure 5:**
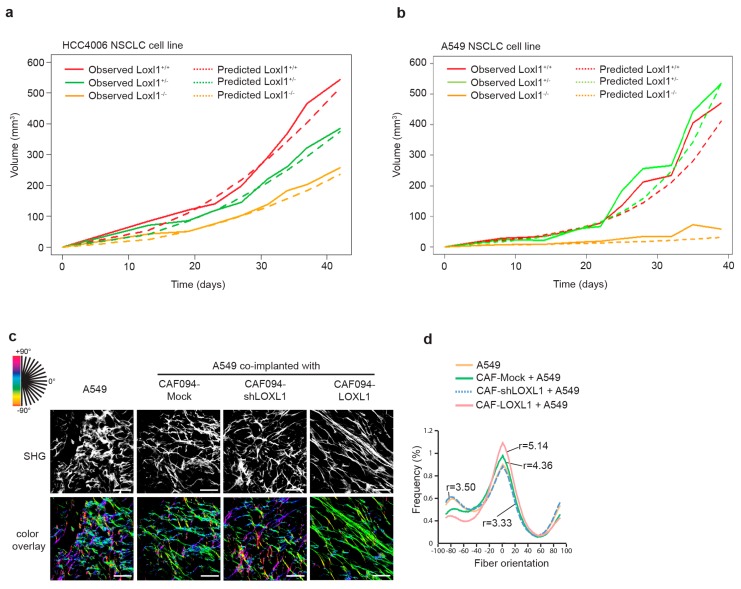
Tumorigenicity of NSCLC cells is impeded in severe combined immune deficient (SCID) mice with a homozygous-deleted Loxl1 genotype. A total of 2 × 10^6^ of HCC4006 lung adenocarcinoma cells (**a**) or A549 lung adenocarcinoma cells (**b**) were implanted subcutaneously into the flank of SCID mice with normal (+/+), heterozygous (+/−), or homozygous (−/−) deleted Loxl1. Tumor growth was monitored and recorded twice a week. Differences in tumor growth rates of xenografts were analyzed using mixed-effect model estimation (see Tables S4 and S5). (**c**) Effect of LOXL1 on collagen fiber alignment on A549/CAF xenograft lung tumor has been evaluated using second harmonic generation (SHG). SHG pictures were analyzed using OrientationJ. Each oriented structure within the image is colored according to its angle. Dominant color represents dominant alignment direction. Scale bar: 100 µm. (**d**) Distribution of collagen fiber orientation within the different conditions. Peaks of distribution were aligned and the ratio between the peak and baseline of each curve was calculated to give the degree of alignment. Statistics were performed using Fisher’s test (27 pictures analyzed per condition, see Table S6).

We have previously demonstrated that integrin α11, in addition of promoting metastasis, supported NSCLC tumor growth [20,21]. Since the role of integrin α11 in NSCLC may require the contribution of LOXL1, we analyzed the effect of Loxl1 knockout on tumor growth. We subcutaneously injected the HCC4006 NSCLC cell line in Loxl1 knockout (Loxl1^−/−^), heterozygous (Loxl1^+/−^), and wild-type (Loxl1^+/+^) mice and measured tumor growth rate. Loxl1 deletion in mice significantly decreased tumor growth compared to wild-type SCID mice (Figure 5a and Table S4). This finding was further validated with the A549 cell line (Figure 5b and Table S5). Analysis of the tumor volume of HCC4006 (*n* = 11–16) and A549 (*n* = 5–7) tumor xenografts in Loxl1^+/+^, Loxl1^+/−^, and Loxl1^−/−^ mice at the experimental endpoint is presented in Supplementary Figure S3.

To investigate whether LOXL1-mediated collagen matrix alignment may have a role in tumor growth, we collected xenograft tumors formed by subcutaneous co-implantation into SCID mice of the A549 NSCLC cell line with the different CAFs with varying expression of LOXL1. We observed that A549/CAF094-LOXL1 xenograft tumors with LOXL1 overexpression demonstrated higher collagen fiber alignment compared to A549/CAF094-Mock xenograft tumors with LOXL1 basal expression (*r* = 5.14 and *r* = 4.3, respectively; *p* = 0.049; Figure 5c,d and Table S6). LOXL1-knockdown xenograft tumors (A549/CAF094shLOXL1) and xenograft tumors formed by A549 alone displayed similar weak collagen organization (*r* = 3.5, *p* = 0.011 and *r* = 3.33, *p* = 0.032, respectively).

## 3. Discussion

Our previous work has suggested a link between integrin α11 and LOXL1 [20]. In the present study we showed that integrin α11 regulated LOXL1 expression in NSCLC CAFs. Furthermore, secretion of LOXL1 by CAF in the TME mediated collagen fiber alignment to support NSCLC tumor growth and tumor cell invasion.

We showed that inactivation of integrin α11 in fibroblast decreased the expression of LOXL1, while overexpression of integrin α11 enhanced its expression. This indicates that integrin α11 regulates LOXL1 expression. Integrin α11 promoter activity is regulated through the TGF-β signaling pathway [26,27]. Although TGF-β has also been reported to regulate LOXL1 in stromal cells [28,29], there have been no promoter analyses associated with these data. According to our data it is, thus, possible that integrin α11 acts as an intermediary between TGF-β and LOXL1 in these studies. It is interesting to note that LOXL1 could regulate Smad2/3 phosphorylation [29], thus establishing a feedback loop.

Cross-linked collagen has been shown to play a role in collagen gel contraction, which reflects collagen remodeling [30]. Furthermore, inhibition of lysyl oxidases using β-aminopropionitril altered collagen remodeling in a wound-healing model in rabbit [31] and reduced tissue contraction in a wound repair model in rat [32]. Here, we observed that collagen remodeling was dependent on LOXL1 expression. However, how collagen cross-linking contributes to collagen matrix contraction is still unclear. Integrins are involved in matrix remodeling and integrin α11 was demonstrated to be the major collagen receptor on fibroblasts that contributes to collagen remodeling [26]. We assume that LOXL1 increased collagen matrix stability, which in turn enhanced the transmitted force through attachment of integrin to the matrix, thus increasing cell contractility and collagen reorganization. We believe that the observed collagen remodeling by the C2C12-LOXL1 cells, in the absence of integrin α11, is dependent on LOXL1 and is mediated by other integrins that contribute to cell–collagen interactions [33].

Collagen remodeling is an important feature of the TME that promotes metastasis [34,35]. Metastatic lung cancer has been shown to have increased linearity of collagen fibers and organization that correlated with increased expression of the lysyl oxidases, LOX and LOXL2 [36]. Although LOX pro-peptide displays tumor suppressor activity [10], LOX family oxidases support metastasis [37]. In NSCLC, high LOX expression is associated with invasion and poor prognosis [38], and targeting the LOX pathway in tumor cells inhibits metastasis [39]. Similarly, high level of LOXL2 is associated with poor prognosis in NSCLC [40] and LOXL2 promotes the formation of a pre-metastatic niche [41]. In contrast, the role of LOXL1 in metastasis is poorly documented. We showed that overexpression of LOXL1 in CAFs significantly increased NSCLC cell invasion, whereas knockdown of LOXL1 decreased this process, as a result of collagen rearrangement.

Previously, we have shown that integrin α11 deficiency inhibited lung tumor growth due to the lack of tissue stiffness that usually accompanies lung tumor formation [20]. Here, we demonstrated that Loxl1 deletion in mice inhibited NSCLC tumor growth. It has been reported that matrix cross-linking that occurs in the TME increases the stiffness of matrix surrounding tumor cells [3]. Furthermore, Paszek et al. showed that the matrix rigidity promotes integrin clustering in breast tumor cells that in turn increases the growth factor-dependent ERK activation to support tumor growth [42]. In NSCLCs, matrix stiffness may regulate PD-L1 expression, which leads to immune response evasion and tumor growth [43]. It also regulates cross-talk metabolism between CAFs and tumor cells to support tumor growth [44].

In our study we have found pro-tumorigenic expression of LOXL1 in the CAFs but not in NSCLC cell lines. Wu et al. have shown that LOXL1 was epigenetically silenced in bladder cancer cells [45]. Reintroduction of LOXL1 expression in these cells promotes tumor suppressor activity, suggesting that the role of LOXL1 in tumor progression could depend on the tumor and the cell type in which it is expressed. Interestingly, in addition to matrix cross-linking, LOX and LOXL2 were found in cell nucleus to regulate gene transcription [46]. For instance, LOXL2 contributes to the stabilization of transcription factor Snail1 to promote breast tumor progression [47]. The translocation of LOXL1 in the nucleus has not yet been investigated, thus a role of LOXL1 in transcription regulation that supports tumorigenesis cannot be excluded.

**Scheme 1:**
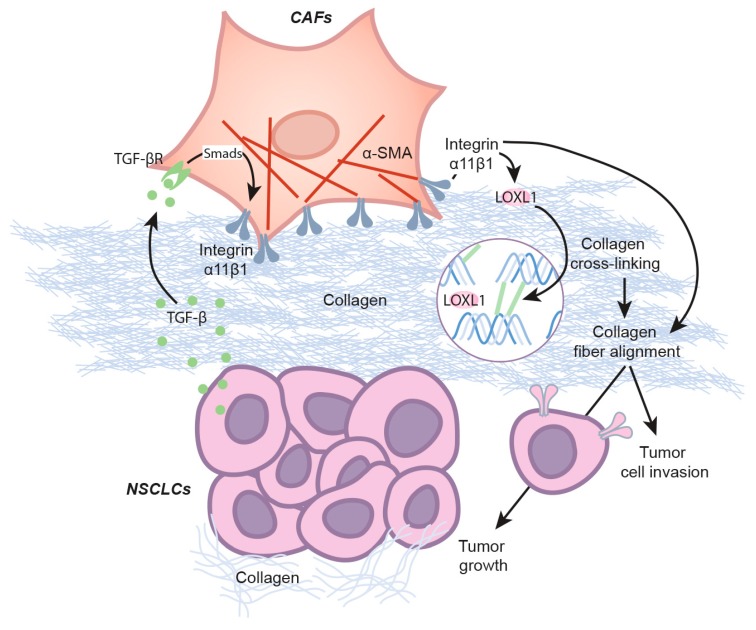
LOXL1 in NSCLC tumorigenicity. NSCLC cells secrete TGF-β that promotes integrin α11 expression in CAFs through the Smad signaling pathway. Integrin α11, in turn, induces LOXL1 expression in CAFs. Once secreted, LOXL1 cross-links collagen fibers that enhance integrin-mediated collagen matrix remodeling. As part of the remodeling, alignment of collagen fibers supports tumor growth and tumor progression.

The results of our study provide direct evidence to support the regulatory role of integrin α11 in LOXL1 gene expression and indicate the important role of LOXL1 as a stroma-specific gene in the progression of NSCLC (Scheme 1).

## 4. Materials and Methods

### 4.1. Correlation of LOXL1 and Integrin α11 Gene Expression in NSCLC Patients

Datasets used for the correlation of LOXL1 and integrin α11 gene expression were the NSCLC dataset from our group at University Health Network (UHN, *n* = 128; GSE50081) [48], Sungkyunkwan University of South Korea (SKKU, *n* = 63; GSE8894) [49] and The Cancer Genome Atlas (TCGA, *n* = 517) [50]. Gene expression analyses were carried out using Affymetrix U133A Plus 2 microarray in the UHN and SKKU dataset, and RNASeq Version 2 RSEM in TCGA dataset. The association of LOXL1 and integrin α11 gene expression was tested using Spearman rank correlation analysis.

### 4.2. Animals and Cell Lines

Severe combined immune deficient (SCID) mice were bred on-site (Animal Resource Centre) at the Princess Margaret Cancer Centre (Toronto, ON, Canada). All manipulations were performed under sterile conditions in a laminar flow hood, in accordance with protocols approved by the Institutional Animal Care Committee (animal use protocol (AUP) ID: 794, approved date: 7 May 2018). The Loxl1-deficient mice [7] were purchased from The Jackson Laboratory. Initially, the Loxl1 heterozygous (+/−) mice were in a homogenous C57BL/6 background. Subsequently, they were bred with the BALB/c severe combined immune deficient (SCID) mice, obtained from the Princess Margaret Cancer Centre, for six generations, resulting in Loxl1^+/−^ mice with a SCID homozygous autosomal recessive mutation. Inter-crossing of heterozygous mice resulted in homozygous viable and fertile offspring with an expected Mendelian ratio. Because it has been previously reported that Loxl1^−/−^ females develop pelvic prolapse post-partum, due to the role of Loxl1 in elastogenesis, only heterozygous females were used for breeding.

NCI-H661, HCC4006, and A549 cell lines were obtained from the American Type Culture Collection and were cultured in Roswell Park Memorial Institute (RPMI) 1640 media supplemented with 10% fetal bovine serum (FBS). A list of 30 NSCLC cell lines used in Figure 1b is provided in Supplementary Table S7. Primary human carcinoma-associated fibroblasts were isolated from NSCLC resection specimens, as described in [1,2]. Patient demographics, tumor stage, and pathological diagnosis for 20 tumors from which these CAFs have been isolated [2] are provided in Supplementary Table S8. Additionally, CAF094 were previously telomerase reverse transcriptase (hTERT)-immortalized [1]. CAF cells were cultured in Dulbecco’s modified Eagle’s medium (DMEM) supplemented with 10% FBS. Mouse embryonic fibroblasts, MEF α11^+/+^, MEF α11^−/−^ and MEF α11^−/−^ KI [22], and murine C2C12 myoblasts, wild-type and C2C12-α11 [24], were obtained from Dr. Donald Gullberg (University of Bergen, Bergen, Norway).

### 4.3. Generation of Stable LOXL1 Knockdown and Overexpression

LOXL1 gene expression was stably knocked down using short hairpin RNA (shRNA) constructs. The shRNAs were obtained from the RNAi Consortium of University of Toronto. LOXL1 was stably knocked down by the shLOXL1 vector#1 with a target sequence 5′- ACGTGGTGAGATGCAACATTC and shLOXL1 vector#2 with a target sequence 5′- CGCTACGTTTCTGCAACAAAC. Human full length LOXL1 cDNA was sub-cloned into a pLKO lentiviral expression vector. The empty vector construct was used as control. Lentiviruses were prepared by transfecting three plasmids into 293T cells: (i) pMDLg/pRRE, the vesicular stomatitis virus (VSV-G) envelope plasmid pCMV-VSG, (ii) the rev-expressing plasmid pRSV-Rev, and (iii) one of either of the gene transfer vectors: pLKO-shLOXL1 vectors, pLKO-LOXL1, or the empty control vector. Stocks were stored frozen at −80 °C. CAFs transduced with these constructs were selected by 1 µg/mL puromycin.

### 4.4. Quantitative Real-Time PCR

Total RNA was isolated from cultured cells using the Qiagen RNEasy Kit (Qiagen, Venlo, Netherlands). Total RNA was reverse-transcribed to synthesize 1 to 3 µg of cDNA, using Superscript III^®^ (Life Technologies, Burlington, ON, Canada). The cDNA was diluted and 10 ng was used for each quantitative PCR reaction performed by CFX96 Touch™ RT-PCR Detection System (BioRad, Mississauga, ON, Canada), using 2X SYBR Green master mix (Life Technologies). For primer list, please refer to Supplementary Table S9.

### 4.5. Western Blot Analysis

For detection of secreted LOXL1, cells were grown to confluence in 2 × 15 cm culture plates and incubated in serum-free DMEM medium for 48 h. The conditioned media was then collected, passed through a 0.45 µm filter, frozen at −80 °C overnight, and concentrated through lyophilization. Secreted protein powder was reconstituted in water. Protein samples were separated by sodium dodecyl sulfate/polyacrylamide gels and transferred onto polyvinylidene fluoride (PVDF) membranes using the Trans-Blot^®^ Turbo™ Transfer System (BioRad). Membranes were blocked with 5% non-fat dry milk for 1 h at room temperature and then incubated with primary antibodies to rabbit anti-human/mouse LOXL1 (Santa Cruz, Dallas, TX, USA) at 4 °C overnight. Visualization used horseradish peroxidase-conjugated anti-rabbit secondary antibody (Cell signaling, Beverly, MA, USA) and ECL-Plus blotting substrate detection kit (GE Healthcare Life Sciences, Mississauga, ON, Canada). After developing, the membrane was stained in Coomassie blue solution (Coomassie blue R-250 0.1%, isopropanol 25% and acetic acid 10%; distaining solution: methanol 50% and acetic acid 7%) and was used as loading control.

### 4.6. Fibroblast-Populated Collagen Lattice Culture

Collagen gels were prepared as follow: Nine volumes of 4 mg/mL rat tail type I collagen (Advanced Biomatrix, San Diego, CA, USA) were mixed with 1 volume of neutralization solution (Advanced Biomatrix) and 12 volumes of 2× serum-free DMEM medium. CAFs were trypsinized and incorporated in the collagen solution at a final density of 2.5 × 10^5^ cells/mL. Five hundred microliters of the cell/collagen mixture was added into each well of a 24-well plate and allowed to polymerize for 1 h at 37 °C. DMEM supplemented with 10% of serum was added on top of each gel. Twelve days later, collagen gel was released and diameter was measured. Acellular collagen gels were used as negative control.

### 4.7. Second-Harmonic Generation Microscopy

Collagen second harmonic generation (SHG) images were taken with a Zeiss LSM 710 NLO two-photon microscope, equipped with a Coherent Chameleon Ti:Sapphire femtosecond pulsed laser. A 20× (NA = 1.0) water immersion objective lens was used. An excitation wavelength of 840 nm was used so that the SHG signal would be generated and detected at exactly one-half of the excitation wavelength (i.e., 420 nm). Unstained fixed and deparaffinized 10 µm tissue sections or whole collagen gels were visualized and images of 425 × 425 µm regions were taken.

### 4.8. Fibril Orientation Distribution Analysis

To measure the degree of waviness or alignment and orientation of collagen, distribution of local collagen fiber orientation within images was assessed based on methodology published by Rezakhaniha et al. [51]. Briefly, an ImageJ plug-in, OrientationJ, was used. The local orientation and isotropic properties of pixels were derived from structure tensors, which are commonly used in the field of image processing. Tensors were evaluated for each pixel of an input image by computing the continuous spatial derivatives in the *x* and *y* directions using a cubic B-spline interpolation to obtain the local predominant orientation. Graphical outputs show a hue-saturation-brightness (HSB) color-coded map indicating the angles of the oriented structures within the image. Orientation distribution peaks were then aligned before Fisher’s exact test for statistical significance across distributions. The shape of the distribution indicated the degree of alignment within the image, where wide and broad shapes suggested little coherency in alignment, and tight peaks with small standard deviations implied aligned structures. The ratio between the peak and baseline of each curve was calculated to give the degree of alignment.

### 4.9. Organotypic Cell Co-Culture

Organotypic cultures were prepared as follows: Nine volumes of rat tail collagen I (3.8 mg/mL; Advanced BioMatrix; San Diego, CA, USA), 1 volume of neutralization solution (Advanced BioMatrix), 1.2 volumes of Matrigel^®^ (9.8 mg/mL, Corning^®^; Corning, NY, USA), and 7.3 volumes of 2× serum-free DMEM medium were mixed. CAF cells (5 × 10^5^ cells/mL) were resuspended in the collagen/Matrigel^®^ solution and 900 µL of this mixture were pipetted into each well of a 24-well plate. DMEM media, supplemented with 10% FBS, was added on top of the gels, once collagen had polymerized, and was changed every 2–3 days. To allow the CAF cells to remodel the matrix, they were grown within collagen/Matrigel gels for 5 days. On day 5, NCI-H661 tumor cells (5 × 10^5^ cells/900 µL) were seeded on top of each gel and allowed to adhere to collagen gel surface overnight. The following day, the gels were carefully detached from the wells and were placed onto collagen-coated nylon NET filters (Millipore, Billerica, MA, USA), supported by metal grids. The grids were placed into 60-mm plates and RPMI 1640 media, supplemented with 10% FBS, was added to reach the underside of each metal grid, establishing an air–liquid interface. The media was changed every 2–3 days for the next 21 days.

### 4.10. Immunostaining

Organotypic cell cultures were fixed overnight in 4% paraformaldehyde and 0.25% glutaraldehyde at 4 °C and processed to paraffin blocks. Serial section (5 µm thick) from paraffin blocks were stained for hematoxylin and eosin (H&E) and immunostained for anti-Ep-CAM antibody (Ber-EP4).

### 4.11. Subcutaneous Tumorigenicity Assay

Loxl1 SCID mice were bred on-site at the Ontario Cancer Institute (OCI) animal facility. All manipulations were performed under sterile conditions in a laminar flow hood, in accordance with protocols approved by the OCI Animal Care Committee. Tumor cells (2 × 10^6^) were injected subcutaneously into the abdominal flanks of 4- to 6-week old Loxl1^+/+^, Loxl1^+/−^ and Loxl1^−/−^ SCID mice. For co-injection, 2 × 10^6^ A549 tumor cells with or without 2 × 10^6^ fibroblast cell lines were implanted subcutaneously into the abdominal flanks of the mice. Tumor growth was assessed every two days by measuring tumor length and width with calipers. Tumor volume was calculated using the formula (length × width^2^)π/6. At sacrifice, tumor mass was recorded, and portions of tumors were either snap-frozen in liquid nitrogen or fixed in 10% neutral buffered formalin for histological processing. Predicted log tumor volumes were calculated using mixed effects modeling with the main effects Day, Loxl1 type, and their interaction, and a random intercept for each mouse. The predicted log tumor volumes were transformed to predict the tumor volume.

### 4.12. Statistical Analysis

Differences in tumor growth rates of xenografts were tested using mixed effect model estimation. Fisher’s exact test was used for analysis of fibril orientation distribution. Collagen matrix contraction was analyzed using ANOVA followed by Bonferroni post-test. Mann–Whitney test was used for RT-qPCR, Western blotting, and invasion experiments.

## 5. Conclusions

Our findings demonstrate that stromal LOXL1, under regulation of integrin α11, is a determinant factor of NSCLC tumorigenesis and may be an interesting therapeutic target for this disease.

## Supplementary Materials

The following are available online at https://www.mdpi.com/2072-6694/11/5/705/s1, Figure S1: Spearman correlation between integrin α11 and LOXL1 gene expression in NSCLC squamous cell carcinoma was analyzed from RNA-Seq data of the TCGA patient dataset (*n* = 501) or microarray data of UHN (*n* = 43, GSE50081) and SKKU (*n* = 75, GSE8894) patient dataset, Figure S2: Effect of LOXL1 overexpression in C2C12-mediated collagen remodeling. (a) Analysis of human LOXL1 expression in C2C12s by RT-qPCR. (b) C2C12 cell lines were embedded in attached collagen gel and collagen gel diameter was measured 12 days later. Collagen gel contraction was assessed photographically. Scale bar: 1.5 mm. Statistics were performed using Mann–Whitney (**, *p* < 0.01, n.s., not significant), Figure S3: Analysis of tumor volume at day 40 of *HCC4006* (*n* = 11–16) and *A549* (*n* = 5–7) xenografts in *Loxl1^+/+^*, *Loxl1^+/−^*, and *Loxl1^−/−^* mice. The differences between these groups were tested using Mann–Whitney test, Figure S4: Full-size Western blots of Figure 2b. Protein extracts from indicated cells were transferred to a PVDF membrane, and the membrane was blotted with antibodies to LOXL1. Molecular weight marker (MW) was used and sizes of the bands were indicated. (a) Developed film. (b) Developed PVDF membrane stained with Coomassie blue. (c) Overlay of film and blot membrane. The expected size for LOXL1 is around 69 kDa, Table S1: Spearman correlation between ITGA11 and LOX family member expression in three independent lung adenocarcinoma patient cohorts from UHN (*n* = 128; GSE50081), SKKU (*n* = 63; GSE8894), and The Cancer Genome Atlas (TCGA, *n* = 517), Table S2: Statistical analysis of collagen fiber alignment in CAF-populated collagen matrix (based on Figure 3a,b). Fisher’s exact test was used for analysis of fibril orientation distribution, Table S3: Statistical analysis of collagen fiber alignment in Loxl1 knockout and wild-type skin mice (based on Figure 3c,d). Fisher’s exact test was used for analysis of fibril orientation distribution, Table S4: Statistical analysis of HCC4006 lung adenocarcinoma tumor growth in Loxl1^+/+^, Loxl1^+/−^, and Loxl1^−/−^ mice (based on Figure 5A). The comparison between groups was performed within the mixed effect modeling. The mouse was considered a random effect while the time and the genetic group and their interaction were the fixed effects. The volume of the tumor was the dependent variable. The residuals were inspected, and a square root transformation was applied to the volume to obtain residuals distributed normally and to eliminate the heteroscedasticity, Table S5: Statistical analysis of A549 lung adenocarcinoma tumor growth in Loxl1^+/+^, Loxl1^+/−^, and Loxl1^−/−^ mice (based on Figure 5B). The comparison between groups was performed within the mixed effect modeling. The mouse was considered a random effect, whereas the time and the genetic group and their interaction were the fixed effects. The volume of the tumor was the dependent variable. The residuals were inspected, and a log transformation was applied to the volume to obtain residuals distributed normally and to eliminate the heteroscedasticity. Models were fit starting when the tumor volume was above zero (generally day 6), Table S6: Statistical analysis of collagen fiber alignment in A549/CAF xenograft model (based on Figure 5c,d). Fisher’s exact test was used for analysis of fibril orientation distribution, Table S7: List of NSCLC established cell lines used in Figure 1b and their associated histology, Table S8: Patient demographics, tumor stage, and pathological diagnosis for 20 tumor-isolated CAFs used in Figure 1b, Table S9: RT-qPCR primer sequences.
