# Supplementary material for: LOXL1 Is Regulated by Integrin α11 and Promotes Non-Small Cell Lung Cancer Tumorigenicity
Source: Cancers (Basel). 2019 May 22;11(5):705. doi: 10.3390/cancers11050705 (PMC6562909; doi:10.3390/cancers11050705)
Supplement: Supplementary file 1 [file cancers-11-00705-s001.pdf]

Supplementary Material

LOXL1 Is Regulated by Integrin α11 and Promotes Non-Small Cell Lung Cancer Tumorigenicity

Cédric Zeltz, Elena Pasko, Thomas R. Cox, Roya Navab and Ming-Sound Tsao

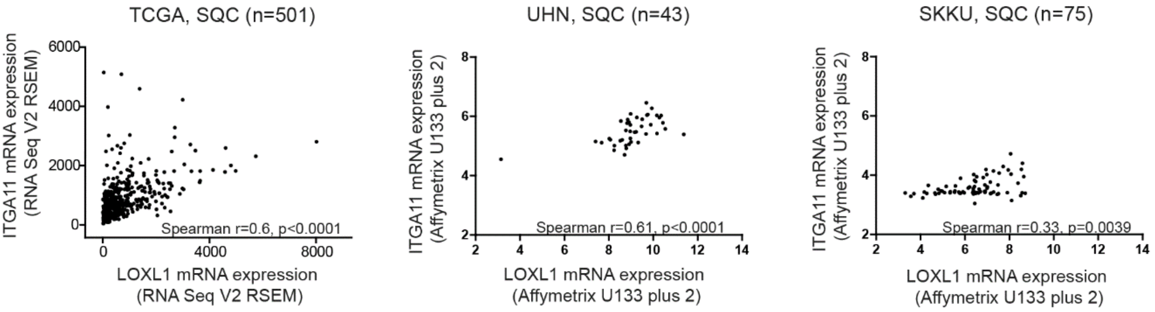

Figure S1. Spearman correlation between integrin α11 and LOXL1 gene expression in NSCLC squamous cell carcinoma was analyzed from RNA-Seq data of the TCGA patient dataset (n = 501) or microarray data of UHN (n = 43, GSE50081) and SKKU (n = 75, GSE8894) patient datasets.

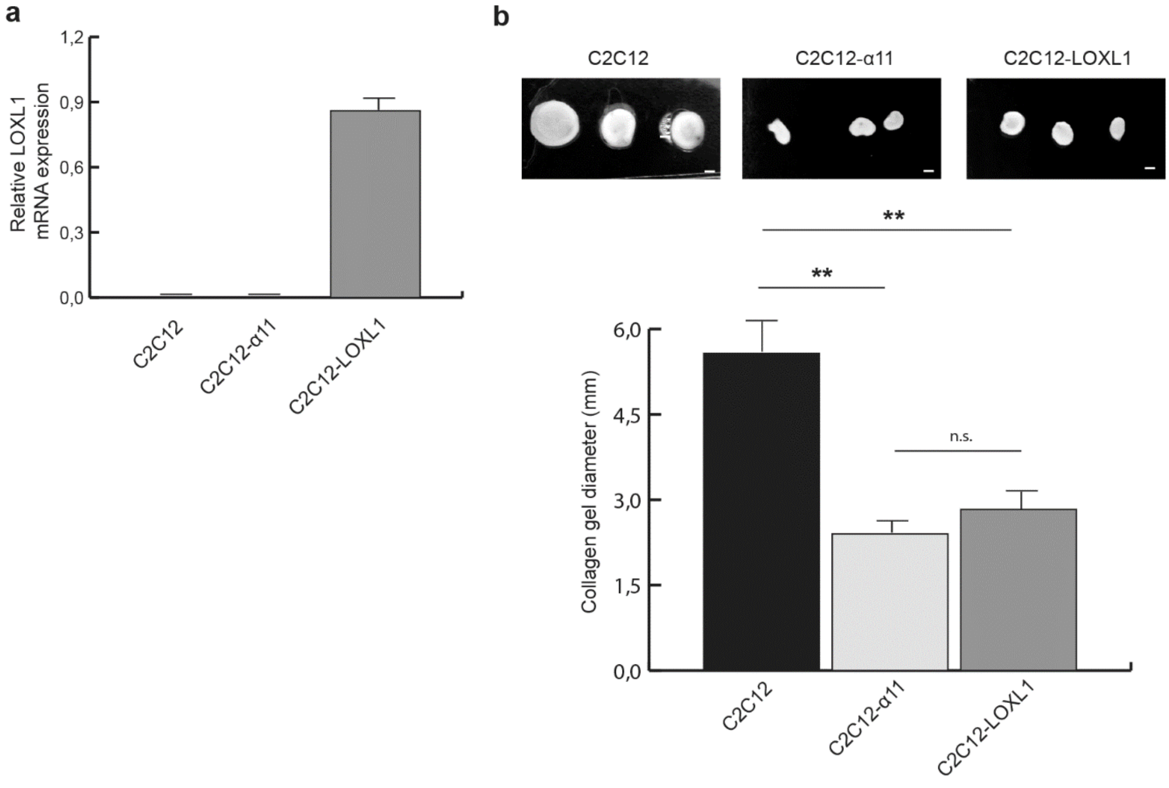

Figure S2. Effect of LOXL1 overexpression in C2C12-mediated collagen remodeling. (**a**) Analysis of human LOXL1 expression in C2C12s by RT-qPCR. (**b**) C2C12 cell lines were embedded in attached collagen gel and collagen gel diameter was measured 12 days later. Collagen gel contraction was assessed photographically. Scale bar: 1.5mm. Statistics were performed using Mann–Whitney (**, $p < 0.01$, n.s., not significant).

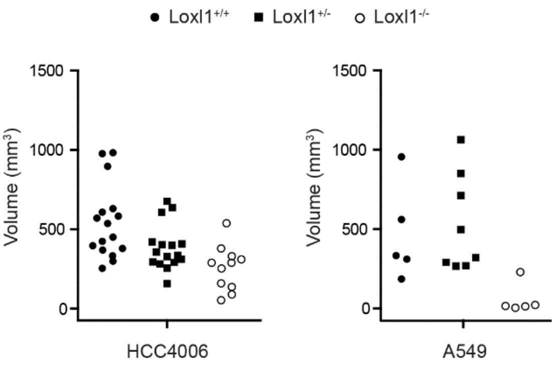

HCC4006

	Mann-Whitney *p*-value
Loxl1+/+ *vs* Loxl1+/-	0.0479
Loxl1+/+ *vs* Loxl1-/-	0.0009
Loxl1+/- *vs* Loxl1-/-	0.0248

A549

	Mann-Whitney *p*-value
Loxl1+/+ *vs* Loxl1+/-	0.9433
Loxl1+/+ *vs* Loxl1-/-	0.0159
Loxl1+/- *vs* Loxl1-/-	0.0016

Figure S3. Analysis of tumor volume at day 40 of HCC4006 (n = 11–16) and A549 (n = 5–7) xenografts in Loxl1$^{+/+}$, Loxl1$^{+/–}$, and Loxl1$^{–/–}$ mice. The differences between these groups were tested using Mann–Whitney test.

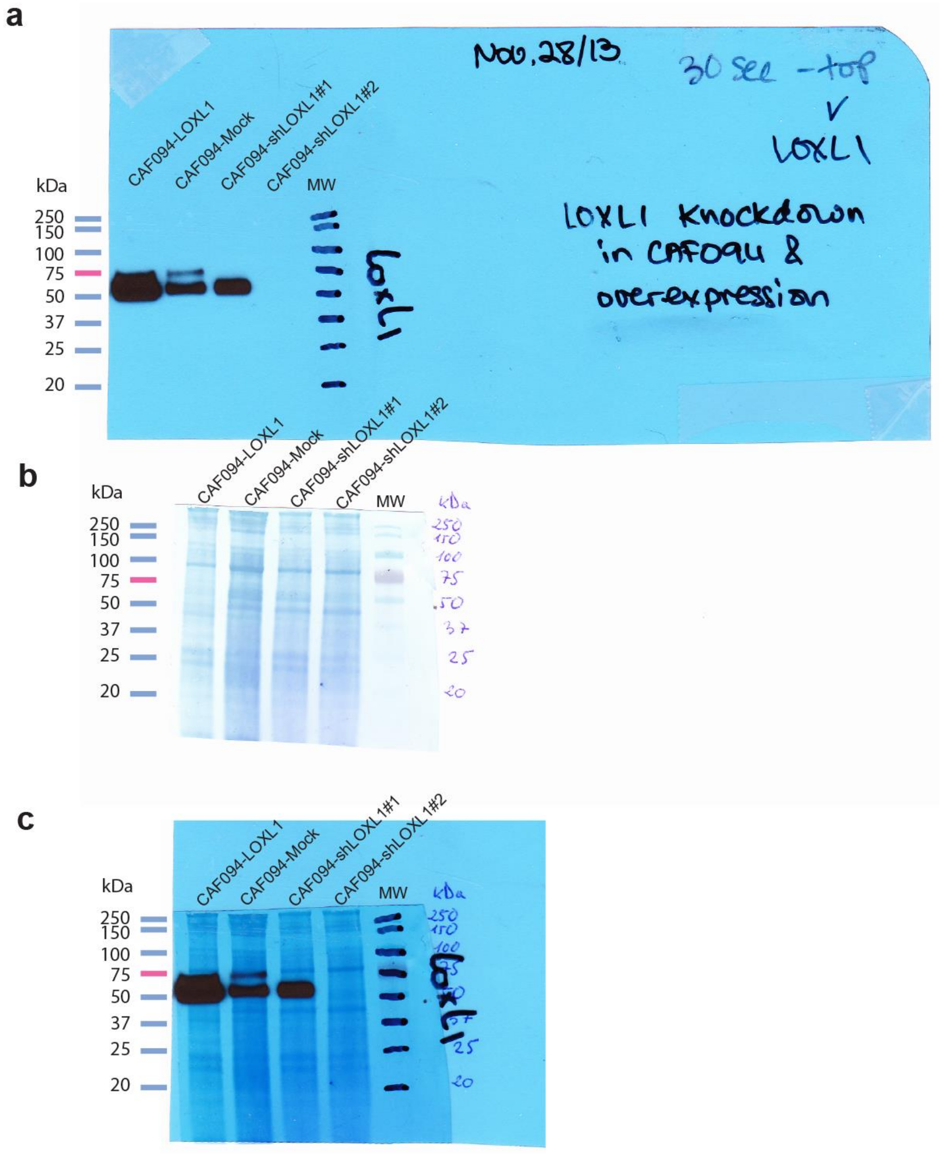

Figure S4: Full-size Western blots of Figure 2b. Protein extracts from indicated cells were transferred to a PVDF membrane, and the membrane was blotted with antibodies to LOXL1. Molecular weight marker (MW) was used and sizes of the bands were indicated. (**a**) Developed film. (**b**) Developed PVDF membrane stained with Coomassie blue. (**c**) Overlay of film and blot membrane. The expected size for LOXL1 is around 69 kDa.

Table S1. Spearman correlation between ITGA11 and LOX family member expression in three independent lung adenocarcinoma patient cohorts from UHN (*n* = 128; GSE50081), SKKU (*n* = 63; GSE8894), and The Cancer Genome Atlas (TCGA, *n* = 517).

Gene	TCGA	UHN	SKKU
LOXL1	0.68 ($p < 0.0001$)	0.78 ($p < 0.0001$)	0.61 ($p < 0.0001$)
LOX	0.61 ($p < 0.0001$)	0.43 ($p < 0.0001$)	0.33 ($p < 0.01$)
LOXL2	0.57 ($p < 0.0001$)	0.65 ($p < 0.0001$)	0.39 ($p < 0.01$)
LOXL3	0.42 ($p < 0.0001$)	0.58 ($p < 0.0001$)	0.46 ($p < 0.001$)
LOXL4	0.19 ($p < 0.0001$)	0.22 ($p < 0.01$)	0 ($p = 0.88$)

Table S2. Statistical analysis of collagen fiber alignment in CAF-populated collagen matrix (based on Figure 3A,B). Fisher's exact test was used for analysis of fibril orientation distribution.

CAF094 Cell Line	Fisher Exact Test *p*-Value
Mock vs. shLOXL1	$1.77e^{-06}$
Mock vs. LOXL1	0.0013
LOXL1 vs. shLOXL1	$4.75e^{-15}$

Table S3. Statistical analysis of collagen fiber alignment in Loxl1 knockout and wild-type skin mice (based on Figure 3C,D). Fisher's exact test was used for analysis of fibril orientation distribution.

Mouse	Fisher Exact Test *p*-Value
$Loxl1^{+/+}$ *vs* $Loxl1^{-/-}$	0.0005

Table S4. Statistical analysis of HCC4006 lung adenocarcinoma tumor growth in $Loxl1^{+/+}$, $Loxl1^{+/-}$, and $Loxl1^{-/-}$ mice (based on Figure 5A). The comparison between groups was performed within the mixed effect modeling. The mouse was considered a random effect, whereas the time and the genetic group and their interaction were the fixed effects. The volume of the tumor was the dependent variable. The residuals were inspected, and a square root transformation was applied to the volume to obtain residuals distributed normally and to eliminate the heteroscedasticity.

Mouse	Difference (Square Root Scale)	95% CI of the Difference (Square Root Scale)	*p*-Value
$Loxl1^{+/+}$ vs. $Loxl1^{-/-}$	0.175	0.129–0.221	<0.0001
$Loxl1^{+/-}$ vs. $Loxl1^{-/-}$	0.088	0.042–0.133	0.0002
$Loxl1^{+/+}$ vs. $Loxl1^{+/-}$	0.088	0.046–0.129	<0.0001

Table S5. Statistical analysis of A549 lung adenocarcinoma tumor growth in Loxl1$^{+/+}$, Loxl1$^{+/-}$, and Loxl1$^{-/-}$ mice (based on Figure 5B). The comparison between groups was performed within the mixed effect modeling. The mouse was considered a random effect, whereas the time and the genetic group and their interaction were the fixed effects. The volume of the tumor was the dependent variable. The residuals were inspected, and a log transformation was applied to the volume to obtain residuals distributed normally and to eliminate the heteroscedasticity. Models were fit starting when the tumor volume was above zero (Generally day 6).

Mouse	Difference (Log Scale)	95% CI of the Difference (Log Scale)	*p*-Value
Loxl1$^{+/+}$ vs. Loxl1$^{-/-}$	0.0438	0.0282–0.0594	<0.0001
Loxl1$^{+/-}$ vs. Loxl1$^{-/-}$	0.0588	0.0447–0.0729	<0.0001
Loxl1$^{+/+}$ vs. Loxl1$^{+/-}$	–0.0151	−0.029–−0.00116	0.0339

Table S6. Statistical analysis of collagen fiber alignment in the A549/CAF xenograft model (based on Figure 5C,D). Fisher's exact test was used for analysis of fibril orientation distribution.

Xenograft Tumor	Fisher Exact Test *p*-Value
A549 vs. Mock	0.033
A549 vs. shLOXL1	0.69
A549 vs. LOXL1	$4.6e^{-5}$
Mock vs. shLOXL1	0.011
Mock vs. LOXL1	0.049
LOXL1 vs. shLOXL1	$8.14e^{-6}$

Table S7. List of NSCLC established cell lines used in Figure 1b and their associated histology.

NSCLC Cell Line	Histology	NSCLC Cell Line	Histology	NSCLC Cell Line	Histology
A549	ADC	H1395	ADC	H2126	LC
DFC1032	ADC	H1437	ADC	H2228	ADC
H23	ADC	H1573	ADC	H2279	ADC
H522	ADC	H1693	ADC	H2405	ADC
H647	ADC	H1792	ADC	H3255	ADC
H650	ADC	H1944	ADC	H4017	LC
H661	LC	H1975	ADC	HCC827	ADC
H838	ADC	H2009	ADC	HCC2935	ADC
H920	ADC	H2073	ADC	HCC4006	ADC
H1373	ADC	H2122	ADC	MGH7	SQC

ADC, adenocarcinoma; LC, large cell; SQC, squamous cell carcinoma.

Table S8. Patient demographics, tumor stage, and pathological diagnosis for 20 tumor-isolated CAFs used in Figure 1b.

PHLC *	Smoking History	Sex	Stage	Histology
094	n.d.	F	III	ADC
448	Ex-Smoker	M	T2N0M0	ADC
453	Smoker	M	T1N0M0	ADC
455	Never	F	T1N0M0	ADC
462	Smoker	F	T2N0M0	SQC
466	Ex-Smoker	M	T2N0M0	ADC
468	Smoker	F	T2N0M0	ADC
472	Never	F	T1N0M0	ADC
476	Ex-Smoker	F	T3N0M0	ADC
479	Smoker	F	T1N0M0	ADC
480	Ex-Smoker	M	T2N0M0	ADC
481	Never	F	T2N0M0	ADC

482	Ex-Smoker	M	T2N0M0	SQC
484	Unknown	F	T1N0M0	ADC
487	Ex-Smoker	M	T4N0M0	SQC
488	Never	F	T1N0M0	ADC
489	Smoker	F	T2N0M0	ADC
491	Ex-Smoker	F	T1N0M0	ADC
492	Smoker	M	T1N0M0	ADC
493	Ex-Smoker	M	T2N0M0	ADC

n.d., not determined, M, male, F, female; * PHLC (primary human lung cancer) numbers refer to 20 hematoxylin and eosin (H&E) slides from NSCLC primary tumors used in CAF cohort.

Table S9. RT-qPCR primer sequences.

Gene	Primer Sequence	
**Mouse**		
Loxl1	Forward	5′- TAGAGTAGTGGGTCTGGAGGC -3′
	Reverse	5′- GGGAGAGGAGCAAAGAAGTGG -3′
Gapdh	Forward	5′- GCAAGGACACTGAGCAAGAGA -3′
	Reverse	5′- ATTATGGGGGTCTGGGATGGA -3′
**Human**		
LOXL1	Forward	5′- GTCGCTACGTTTCTGCAACA -3′
	Reverse	5′- GCTTTGGAAGGGGAGAGATT -3′
ITGA11	Forward	5′- TGCCCTTTTCTCTCACCCATC -3′
	Reverse	5′- CTTTCTTCATCCCTGGCTTGC -3′
RPS13	Forward	5′- GTTGCTGTTCGAAAGCATCTTG -3′
	Reverse	5′- AATATCGAGCCAAACGGTGAA -3′
B2M	Forward	5′- GAGTGCTGTCTCCATGTTTGATGT -3′
	Reverse	5′- AAGTTGCCAGCCCTCCTAGAG -3′

© 2019 by the authors. Licensee MDPI, Basel, Switzerland. This article is an open access article distributed under the terms and conditions of the Creative Commons Attribution (CC BY) license (http://creativecommons.org/licenses/by/4.0/).
